# The Mitochondrial Genome of the Springtail *Semicerura bryophila* (Collembola): New Data Call into Question the Relevance of the Subfamilies of the Isotomidae

**DOI:** 10.3390/genes16030315

**Published:** 2025-03-06

**Authors:** Zhijng Xie, Mingxin Zheng, Yueying Li, Shiyu Du, Ruslan Saifutdinov, Mikhail Potapov, Xin Sun, Donghui Wu

**Affiliations:** 1 Key Laboratory of Vegetation Ecology, Ministry of Education, Northeast Normal University, Changchun 130024, China; xiezhijing@nenu.edu.cn (Z.X.); mingxinzheng@nenu.edu.cn (M.Z.); liyy654@nenu.edu.cn (Y.L.); 2 State Environmental Protection Key Laboratory of Wetland Ecology and Vegetation Restoration, School of Environment, Northeast Normal University, Changchun 130024, China; 3 College of Plant Protection, Nanjing Agricultural University, Nanjing 210095, China; zjjhdsy@126.com; 4 A.N. Severtsov Institute of Ecology and Evolution, Russian Academy of Sciences, 119071 Moscow, Russia; sairusair@yandex.ru; 5 Moscow State Pedagogical University, 119992 Moscow, Russia; mpnk-abroad@yandex.ru; 6 Key Laboratory of Urban Environment and Health, Institute of Urban Environment, Chinese Academy of Sciences, Xiamen 361021, China; xsun@iue.ac.cn; 7 Key Laboratory of Wetland Ecology and Environment, State Key Laboratory of Black Soils Conservation and Utilization, Northeast Institute of Geography and Agroecology, Chinese Academy of Sciences, Changchun 130102, China

**Keywords:** mt genome, soil invertebrates, soil fauna, phylogenetic analysis, eukaryote biodiversity, hexapoda

## Abstract

**Background**: *Semicerura bryophila* Potapov & Sun, 2020 is a soil-dwelling springtail belonging to the family Isotomidae. The phylogenetic relationships among species of this group remain controversial due to a lack of molecular data. Therefore, in this study, we sequenced the mitochondrial genome of *S. bryophila*, analyzed the characterization of the mitochondrial genome, and investigated the phylogenetic relationships of Isotomidae. **Methods**: The mitochondrial genome of *S. bryophila* was sequenced and assembled. We analyzed the sequence length, nucleotide composition, and evolutionary relationships within the Isotomidae family, incorporating data from twelve previously published mitochondrial genomes. **Results**: The length of the *S. bryophila* mitogenome is 15,247 bp and comprises 13 protein-coding genes, 22 tRNAs, and two *rRNA*s, arranged in a typical order. Its base composition is as follows: A = 38.05%, T = 33.64%, G = 10.17%, and C = 15.03%. Phylogenetic analysis based on the mitogenome revealed that the monophyly of Isotomidae and the paraphyletic grouping of *Semicerura* and *Folsomotoma*, supporting their closer relationship with the subfamily Anurophorinae rather than to Isotominae. The analysis validated subfamily Anurophorinae, identified Pachyotominae as a part of Anurophorinae, and suggested that Isotominae is paraphyletic. **Conclusions**: The present study provides valuable mitochondrial information for the classification of *S. bryophila* and offers new insights into the taxonomic and evolutionary studies within the genus *Semicerura*.

## 1. Introduction

Collembola (springtails) are a diverse, abundant, and widespread group that plays a key role in terrestrial ecosystem processes, including carbon and nitrogen cycling, soil microstructure formation, and plant litter decomposition [1,2,3]. Over 9000 species have been described worldwide although it is difficult to give an exact figure as there are many species yet to be discovered [1]. Collembola have an extensive global distribution [4], they are found on every continent, including Antarctica, demonstrating their ability to inhabit a wide range of environments, from tropical forests to polar regions. Their presence in Antarctica is particularly notable, as it represents one of the most extreme and inhospitable environments for life.

The family Isotomidae Börner, 1913 [5], is one the most abundant and widespread in virtually any habitat of the Palaearctic region [6] with 1484 species recorded worldwide (as of 26 February 2025) [4]. Currently, three subfamilies are recognized within the family: Isotominae, Anurophorinae, and Pachyotominae. Among these, the genus *Semicerura* presumably belongs to Isotominae, although it displays several uncommon morphological features for the subfamily [7]. The genus is distributed in North America and East Asia and currently comprises five species [4]. The recently described *S. bryophila* is known to inhabit diverse ecosystems across eastern Asia, ranging from lowlands to alpine regions, and it shows a clear habitat preference for moss [4]. To explore the evolution of the genus within Isotomidae and to validate the subfamilies, the mitochondrial genome of *S. bryophila* Potapov & Sun, 2020 was sequenced, assembled, and annotated, representing the first mitogenome of the genus.

## 2. Materials and Methods

**Figure 1:**
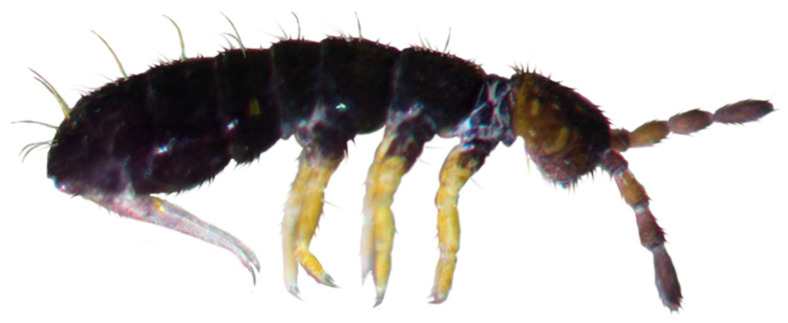
Body coloration of *S. bryophila* Potapov & Sun, 2020. The photograph was captured by Zhijing Xie and has been previously published in Zootaxa [7].

Soil samples containing specimens of *S. bryophila* were collected in May 2015 using a soil corer from the Changbai Mountains in Jilin province, northeast China. Individuals were extracted from soil using Berlese funnels (diameter 20 cm, mesh size 0.84 mm) over ten days without heating, and preserved in 95% ethanol for further analysis. In total approximately 300 specimens were collected from multiple elevations within the Changbai Mountains: 950 m (41.858° N, 127.748° E), 1100 m (41.847° N, 127.798° E), and 1700 m (41.758° N, 127.939° E). At the 1100 m, this elevation appears to be the most favorable for the *S. bryophila*, as it had the highest abundance of specimens collected (approximately 200 individuals, leg. Donghui Wu). This suggests that environmental conditions at this elevation, such as temperature, humidity, vegetation, or other ecological factors, are optimal for their survival and reproduction. Consequently, specimens from this elevation were selected for morphological identification and molecular analyses. The vegetation at 1100 m is characterized by mixed coniferous forests, with dominant tree species including *Pinus koraiensis*, *Picea jezoensis* var. *microsperma*, *Abies nephrolepis*, and *Larix olgensis*. The preserved specimens were imaged using a Zeiss STEMI 508 stereo microscope (Carl Zeiss AG, Oberkochen, Germany) equipped with a DS-Fi1 camera (Figure 1). One specimen was deposited at the Nanjing Agricultural University, Nanjing, China (https://faculty.njau.edu.cn/zhangfeng/zh_CN/ (accessed on 2 February 2025), Prof. Dr. Feng Zhang, fzhang@njau.edu.cn) under the voucher number ‘B4’.

The voucher specimen was cleared in lactic acid and then mounted in cavity and flat slides with Gisin’s liquid and Marc André II solution, respectively. Morphology was studied under a Nikon Eclipse 80i microscope (Nikon Corporation, Tokyo, Japan). Species identification was based on the contrasting coloration (black trunk versus yellow-white legs), the number of spines on the dens, and sparsely ciliated strong macrochaetae [7]. It was identified by comparing it with the holotype of *S. bishopi* Maynard, 1951, kept in the Smithsonian National Museum of Natural History. This comparison focused on key diagnostic characters, including chaetotaxy (e.g., the number and arrangement of chaetae on the body and appendages), spine patterns on the dens (the presence of 2 + 2 short spine-like chaetae in *S. bryophila* versus 1 + 1 in *S. bishopi*), labial palp chaetae (5 basomedian chaetae in *S. bryophila* versus 4 in *S. bishopi*), and other morphological features such as the number of acuminate apical chaetae on Legs I–III (8, 9, 9 in *S. bryophila* versus 8, 8, 8 in *S. bishopi*). To further strengthen the results of identification process, we incorporated molecular tools for verification.

**Figure 2:**
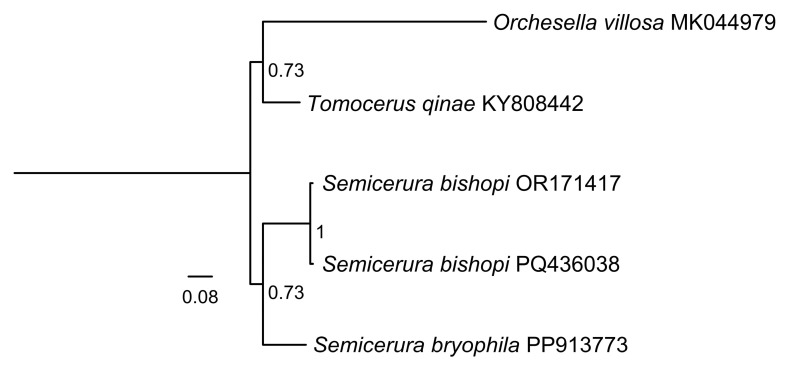
Maximum likelihood phylogenetic tree inferred based cox1 sequences. Maximum-likelihood bootstrap support values are shown in the branches.

Specifically, we downloaded the cox1 sequences of *S. bishopi* from NCBI, amplified and sequenced the corresponding region for our *S. bryophila* specimens, and compared the resulting sequences. Sequences were aligned and manually checked and corrected, resulting in a final 658 bp segment of the Cytochrome c oxidase subunit I (COI) gene, with *Orchesella villosa* and *Tomocerus qinae* used as outgroups. Maximum likelihood tree was conducted using MEGA version X [8] with the Kimura-2 parameter model [9] and bootstrap method with 1000 replicates. The phylogenetic tree illustrates the genetic relationships among the studied specimens (Figure 2), including *S. bryophila* and *S. bishopi*. The tree highlights *S. bryophila* as a distinct lineage, further confirming the genetic distinction between *S. bryophila* and *S. bishopi*, and supporting the morphological findings. This combined approach—morphological and molecular—provides a robust and comprehensive method for species identification, particularly in cases where morphological differences are subtle or where geographical variation may obscure diagnostic traits.

### 2.1. DNA Extraction

DNA was extracted from an individual collected in Changbai Mountains, northeast China (41.847° N, 127.798° E; altitude ca 1100 m; May 2015, leg. Donghui Wu). The sample was deposited at Nanjing Agricultural University (BioSample accession SAMN41817341). DNA extraction was performed using the Ezup Column Animal Genomic DNA Purification Kit (Sangon Biotech, Shanghai, China) following the manufacturer’s protocol. The genomic DNA was stored at −80 °C prior to analysis.

### 2.2. Mitogenome Sequencing, Assembly, and Annotation

The cytochrome c oxidase subunit I (cox1) gene was amplified using primers LCO1490 and HCO2198 [10], following the protocol of Zhang et al. (2014) [11]. PCR products were visualized using 1% agarose gel electrophoresis, then purified and sequenced by Majorbio (Shanghai, China) using the ABI 3730XL DNA Analyzer (Applied Biosystems, Waltham, MA, USA). Sequences were assembled using Sequencher 4.5 (Gene Codes Corporation, Ann Arbor, MI, USA), and aligned using MEGA 7.0 [12]. The resulting cox1 alignment, spanning 658 bp, was used as the seed sequence for the assembly (cox1 accession number: PP913773).

The DNA concentration was quantified using a Qubit 3.0 with the Q33230 Qubit™ 1X dsDNA HS Assay Kit (Thermo Fisher Scientific, Waltham, MA, USA). A DNA pool was created by combining equal concentrations of DNA from the target species with seven other species. Libraries were sequenced with 350 bp insert sizes on the HiSeq X Ten platform (Tianjin Novogene Bioinformatics Technology Co., Ltd., Tianjin, China), producing 150 bp paired-end reads. The average read depth for the assembled mitochondrial genome was 279.02 X. Non-mitochondrial reads were filtered out using NextGenMap 0.5.5 [13] and SAMtools 0.1.18 [14]. Mitogenome assembly was conducted with NOVOPlasty v2.7.0 [15] using the *cox1* sequence as a seed. Chimeric sequences were identified using VSEARCH 2.2.0 [16]. Annotations were performed with the MitoZ v2.4 [17]. The annotated genome sequence was deposited in GenBank under accession numbers: PP915875.

### 2.3. Phylogenetic Analysis

**Figure 3:**
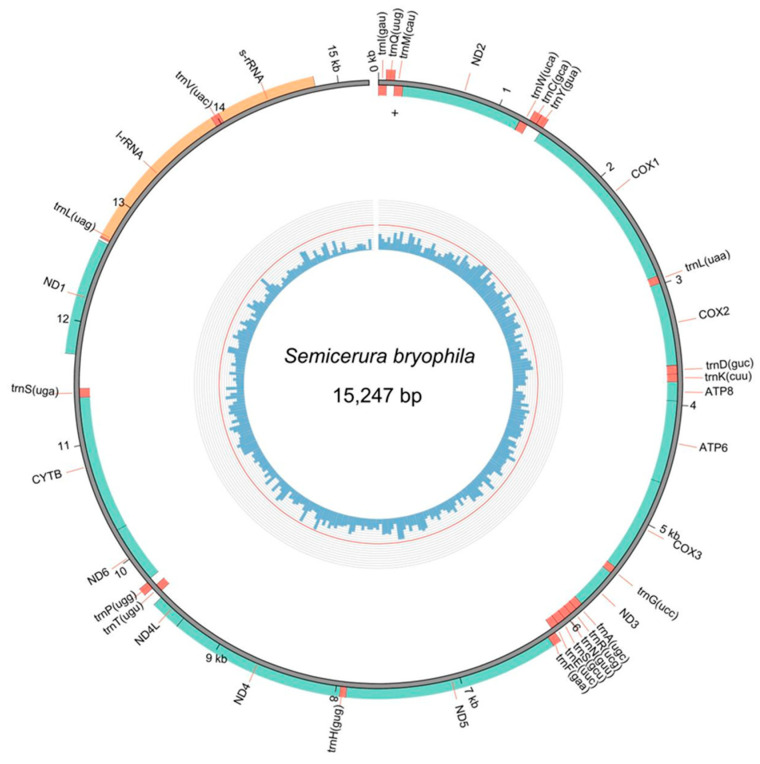
The mitochondrial genome organization of *S. bryophila* Potapov & Sun, 2020 (the accession number PP915875).

All published mitochondrial genes of Isotomidae were included in our analysis to provide novel insights into the family’s classification (Figure 3). Mitochondrial protein-coding gene (PCG) amino acid sequences were aligned with MAFFT v7.394 [18] and trimmed using trimAL v1.4 [19]. Supermatrices of 13 PCGs were constructed using FASconCAT-G v1.04 [20]. Maximum likelihood (ML) phylogenetic analysis on sequences from ten Isotomidae species: five species from the subfamily Isotominae (*Folsomotoma octooculata*, *Isotomurus maculatus*, *Kaylathalia klovstadi*, *Metisotoma macnamarai*, and *S. bryophila*), four from Anurophorinae (*Cryptopygus antarcticus*, *C. terranovus*, *Folsomia candida*, and *Proisotoma minuta*), and one from Pachyotominae (*Paranurophorus simplex*). Two Entomobryoidea species (*Orchesella cincta*, *O. villosa*) and one Tomoceroidea species (*T. qinae*) were used as outgroups. ModelFinder determined models for the supermatrices [21], analyzed in IQ-TREE version 1.6.12 [22] with 1000 replicates for both UFBoot (ultrafast bootstrap approximation) and SH-aLRT (SH-like approximate likelihood ratio) test to assess branch support values [23,24]. The phylogenetic tree was edited using FigTree version 1.4.4 (available at http://tree.bio.ed.ac.uk/software/figtree/, accessed on 2 February 2025).

## 3. Results

### 3.1. Genome Organization and Composition

The mitogenome of *S. bryophila* is 15,247 bp in length (Figure 3), which is comparable to species from the Isotomidae family (Table 1), which typically range from 15,147 bp (*F. candida*) to 15,930 bp (*P. minuta*). Similarly, in related families such as Tomoceridae and Orchesellidae, mitogenome lengths vary from 14,924 bp (*O. villosa*) to 15,728 bp (*O. cincta*), indicating a general consistency in mitogenome size across Collembola. The mitogenome of *S. bryophila* comprises 13 protein-coding genes (PCGs), 22 transfer RNA (*tRNA*) genes, two ribosomal RNA (*rRNA*) genes (Table 2). The base composition is 38.05% adenine, 33.64% thymine, 10.17% guanine, and 15.03% cytosine. Additionally, the sequenced portion lacks the A+T-rich region, although a 436 bp non-coding sequence is present at the junction between the *12S rRNA* and *trnI* genes. Although the sequence is already available in GenBank (accession number PP915875), we were the first to determine the species name and reviewed the published taxonomic [7] and ecological [25] literature related to this species.

### 3.2. Phylogeny of Collembola

**Figure 4:**
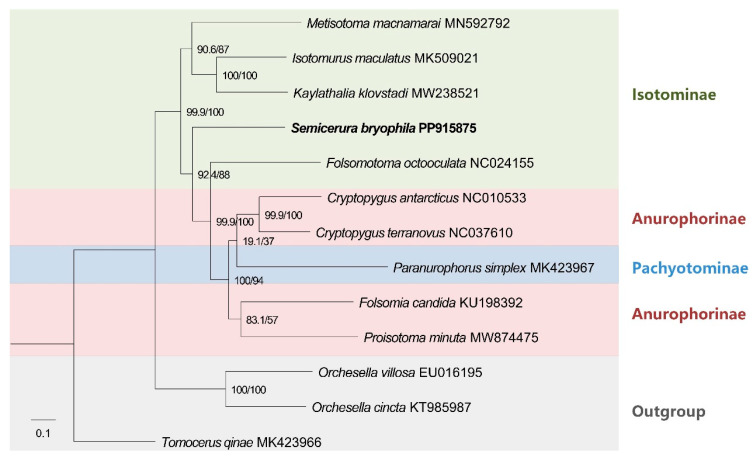
Maximum likelihood phylogenetic tree inferred from partitioned amino acid sequences of 13 PCGs. Support values on nodes indicate SH-aLRT/UFBoot2, respectively. The newly assembled mitogenome of *S. bryophila* was highlighted in bold.

The phylogenetic analysis (Figure 4) confirmed the monophyly of Isotomidae, including the subfamilies Isotominae, Anurophorinae, and Pachyotominae, with Entomobryoidae and Tomoceroidea as outgroups. This finding aligns with the traditional classification of Isotomidae as a distinct family within Collembola.

The subfamily Isotominae appears to be paraphyletic, as *Metisotoma*, *Isotomurus* and *Kaylathalia* clustered together, while *F. octoculata* does not group with them. Instead, *Folsomotoma* shows a closer relationship to Anurophorinae (*C. antarcticus*, *C. terranovus*, *F. candida*, and *P. minuta*). This challenges the traditional classification of Isotominae based on morphological traits. *S. bryophila* clusters outside the core Isotominae group and is more closely related to Anurophorinae and Pachyotominae (*P. simplex*). This finding suggests that *Semicerura* may not belong to Isotominae, despite its morphological similarities, and highlights the need for a re-evaluation of its taxonomic placement.

The subfamily Anurophorinae is validated as a monophyletic group. However, Pachyotominae is nested within Anurophorinae, suggesting that it may not deserve separate recognition as a distinct subfamily. This finding is consistent with recent morphological studies that question the distinctiveness of Pachyotominae.

## 4. Discussion

The first mitogenome of the species *S. bryophila* comprises 13 PCGs genes, 22 tRNA genes, two *rRNA* genes, which is typical for metazoans [37,38]. The mitogenome length of *S. bryophila* (15,247 bp) is consistent with the typical size range observed in Isotomidae and related families of Collembola. This suggests that the mitogenome structure and organization in *S. bryophila* are similar to those of other Collembola, with minor variations likely due to, e.g., species-specific evolutionary adaptations. The comparative data highlight the stability of mitogenome size within Collembola, despite some exceptions such as *P. simplex* (9518 bp), which may reflect unique genomic features or assembly artifacts.

According to the modern and widely accepted classification of Isotomidae [6], the three subfamilies primarily differ in the anatomy of the furca: Isotominae have a long furca with slender, crenulated dens; while in Pachyotominae and Anurophorinae the furca may be middle-sized, short or absent. When present, Pachyotominae exhibit stout, non-crenulated dens. Additionally, the body cuticle is smooth in Isotominae and Anurophorinae but granulated in Pachyotominae.

In our phylogenetic reconstruction, the subfamily Isotominae appears to be paraphyletic, suggesting that its morphological diagnosis should be reconsidered in future studies. The genera *Folsomotoma* and *Semicerura* cluster outside the reference Isotominae (comprising *Metisotoma*, *Isotomurus* and *Kaylathalia*) and are closely related to Anurophorinae (*C. antarcticus*, *C. terranovus*, *F. candida*, and *P. minuta*) and Pachyotominae (*P. simplex*). Rather than forming a distinct clade, *Folsomotoma* and *Semicerura* exhibit a paraphyletic grouping within Anurophorinae, challenging their traditional placement within Isotominae. This moderate relationship is partially supported by morphological evidence, as both *Folsomotoma* and *Semicerura* tend to develop spines on the furca. However, despite *Semicerura* exhibiting several unique and rare morphological traits [7], none of these features are shared with Anurophorinae. In addition, genera within the subfamily Isotominae appear to be largely separated latitudinally, with *Semicerura* predominantly restricted to the Northern Hemisphere, while *Folsomotoma* is found exclusively in the Southern Hemisphere [39].

The paraphyletic grouping of *Semicerura* and *Folsomotoma* aligns with findings from previous molecular studies, which also suggest that Isotominae may not be monophyletic [40,41,42]. Earlier phylogenetic analyses have shown that some genera traditionally placed in Isotominae (e.g., *Folsomotoma*) exhibit closer genetic relationships to Anurophorinae, further challenging the current classification [26]. This finding challenges the current classification of these genera and raises important questions about the morphological and genetic relationships within Isotomidae. Our results suggest that Isotominae may need to be redefined, excluding *Semicerura* and *Folsomotoma*, which appear more closely related to Anurophorinae. Additionally, the morphological traits historically used to define Isotominae (e.g., furca structure) may not be reliable diagnostic characters, as they seem to have evolved convergently in different lineages. Therefore, a revised classification system integrating both morphological and molecular data will be essential to accurately reflect evolutionary relationships within Isotomidae.

The independence of Pachyotominae is not supported by our results—*Paranurophorus* appears as a typical member of Anurophorinae in the phylogenetic tree. This finding is consistent with recent morphological studies showing that Pachyotominae does not consistently exhibit numerous s-setae on the body surface [43]. This character was one of the differentiated characters in primary diagnosis of the family [3,44]. Secondary granulation of body cuticle in Pachyotominae was also previously considered a reliable diagnostic trait until the study by Potapov et al. [45], which demonstrated that the genus *Isotopenola* also exhibits a granulated cuticle, despite belonging to Anurophorinae. However, it is important to note that only one species from Pachyotominae (*P. simplex*) has been sequenced to date in NCBI. This limited representation restricts our ability to draw definitive conclusions about the subfamily’s phylogenetic status. Future work should include additional taxa from Pachyotominae, as well as other underrepresented subfamilies, to better resolve their evolutionary relationships and clarify the taxonomic boundaries within Isotomidae.

## 5. Conclusions

This study represents the first mitogenome of *S. bryophila*. All 37 genes of the *S. bryophila* mitogenome were encoded on the heavy chain, and the gene order of 13 PCGs was completely consistent with that of all known Collembola sequences. To provide novel insights into the family’s classification, all published mitochondrial genes of Isotomidae were included in our analysis. *S. bryophila* is more closely related to the subfamily Anurophorinae than to Isotominae. The monophyly of subfamily Anurophorinae was validated, Pachyotominae was found to be a part of Anurophorinae, and Isotominae appeared to be paraphyletic. The first mitochondrial genome of the springtail *S. bryophila* (Collembola, Isotomidae) challenges the relevance of the current subfamilies within the family. Further analyses incorporating more mitochondrial DNA sequences from these three subfamilies are needed to better resolve the evolutionary relationships, as the current support values are insufficient to draw definitive conclusions. These findings highlight the need for a revised classification system that integrates both morphological and molecular data.

## Figures and Tables

**Table 1:** Information on the mitochondrial genomes of collembolan species used for evolutionary relationship analysis in this study.

Family	Subfamily	Species	Size (bp)	Accession No.	Reference
Isotomidae	Isotominae	*Metisotoma macnamarai*	15,177	MN592792	[26]
Isotomidae	Isotominae	*Isotomurus maculatus*	15,263	MK509021	[27]
Isotomidae	Isotominae	*Kaylathalia klovstadi*	15,485	MW238521	[28]
Isotomidae	Isotominae	*Semicerura bryophila*	15,247	PP915875	This study
Isotomidae	Isotominae	*Folsomotoma octooculata*	15,338	NC024155	[29]
Isotomidae	Anurophorinae	*Cryptopygus antarcticus*	15,297	NC010533	[30]
Isotomidae	Anurophorinae	*Cryptopygus terranovus*	15,352	NC037610	[31]
Isotomidae	Pachyotominae	*Paranurophorus simplex*	9518	MK423967	[32]
Isotomidae	Anurophorinae	*Folsomia candida*	15,147	KU198392	[33]
Isotomidae	Anurophorinae	*Proisotoma minuta*	15,930	MW874475	[34]
Tomoceridae	Tomocerinae	*Tomocerus qinae*	15,045	MK423966	[32]
Orchesellidae	Orchesellinae	*Orchesella villosa*	14,924	EU016195	[35]
Orchesellidae	Orchesellinae	*Orchesella Cincta*	15,728	KT985987	[36]

**Table 2:** Summary of genetic components of *S. bryophila* Potapov & Sun, 2020 mitogenome.

Gene	Start	Stop	Strand	Size (bp)
*trnI* (gau)	1	64	+	64
*trnQ* (uug)	63	130	+	68
*trnM* (cau)	130	199	+	70
*ND2*	200	1198	+	999
*trnW* (uca)	1202	1268	+	67
*trnC* (gca)	1268	1328	-	61
*trnY* (gua)	1331	1392	-	62
*COX1*	1393	2926	+	1534
*trnL* (uaa)	2927	2989	+	63
*COX2*	2990	3673	+	684
*trnK* (cuu)	3676	3746	+	71
*trnD* (guc)	3747	3809	+	63
*ATP8*	3810	3974	+	165
*ATP6*	3968	4648	+	681
*COX3*	4648	5434	+	787
*trnG* (ucc)	5435	5496	+	62
*ND3*	5497	5841	+	345
*trnA* (ugc)	5840	5900	+	61
*trnR* (ucg)	5902	5964	+	63
*trnN* (guu)	5960	6023	+	64
*trnS* (gcu)	6024	6091	+	68
*trnE* (uuc)	6093	6155	+	63
*trnF* (gaa)	6155	6217	-	63
*ND5*	6218	7919	-	1702
*trnH* (gug)	7920	7982	-	63
*ND4*	7983	9354	-	1372
*ND4L*	9357	9623	-	267
*trnT* (ugu)	9641	9702	+	62
*trnP* (ugg)	9703	9764	-	62
*ND6*	9776	10,255	+	480
*CYTB*	10,252	11,388	+	1137
*trnS* (uga)	11,387	11,458	+	72
*ND1*	11,735	12,655	-	921
*trnL* (uag)	12,677	12,741	-	65
*rrn* *L*	12,742	14,003	-	1262
*trnV* (uac)	13,958	14,022	-	65
*rrn* *S*	14,023	14,812	-	790

## Data Availability

The genome sequence data that support the findings of this study are openly available in GenBank of NCBI at (https://www.ncbi.nlm.nih.gov/, accessed on 2 February 2025) under the accession number PP915875. The associated BioProject, SRA and Bio-Sample numbers are PRJNA1123662, SRR31059156 and SAMN41817341.
